# Health disparities and selection bias in obtaining broad consent in a general practitioner setting

**DOI:** 10.3389/fmed.2025.1450274

**Published:** 2025-09-11

**Authors:** Christine Brütting, Konstantin Moser, Marcus Heise, Felix Bauch, Thomas Frese

**Affiliations:** Institute of General Practice and Family Medicine, Center of Health Sciences, Martin-Luther-University Halle-Wittenberg, Halle (Saale), Germany

**Keywords:** BeoNet-Halle, broad consent, general practice, informed consent, health and morbidity-related differences

## Abstract

**Background:**

For research with electronic health records in the outpatient setting, obtaining Broad Consent (BC) is increasingly important. However, the presence of potential selection bias in this context remains unclear. Since 2020, the BeoNet-Halle outpatient database collects patient data from participating general and specialty ambulatory practices’ management systems in Germany, whereby data is obtained anonymously or pseudonymously *via* BC. For clarity, anonymized datasets are routinely extracted for descriptive analyzes, whereas pseudonymized datasets are available only when patients provide BC (details in Methods and Ethics). The primary objective of this study is to compare health related parameters between patients who provided BC and the general practice population.

**Methods:**

This is a single-center, cross-sectional study. From February 2021 to May 2023, patients were asked by a general practitioner or a specially trained member of the joint practice to provide BC. Within the yearly contact group of 2022, we compared patients who provided BC with the reference population (RP) of patients with at least one physician–patient contact during that period in a joint practice including eight eneral ractitioners. Data pertaining to health, morbidity, and health utilization were extracted from the BeoNet-Halle database.

**Results:**

A total of 5,034 patients were analyzed (BC-group: 439 vs. RP group: 4,595). Sex was similar distributed between the groups. In the BC group, patients were slightly older (56.2 vs. 54.1 years), had more physician contacts (15.0 vs. 9.2) and more often at least one chronic condition (76.1 vs. 51.6%) than patients from RP group. Patients were more likely to be referred to at least one other specialist (74.0 vs. 44.4%) and to get at least one drug prescription (89.5 vs. 69.6%).

**Conclusion:**

Differences between BC and the reference population (older age, higher multimorbidity, more contacts, referrals, and drug use) indicate selection processes at the point of consent. Given the single-practice design and descriptive analysis, generalizability beyond similar German group practices is limited and requires validation in multi-site studies.

## Introduction

The utilization of data from outpatient care for scientific research is of great interest as it provides real-world insights into disease prevalence, treatment effectiveness, and healthcare resource utilization across a broad population (1). In Germany, data from social insurance providers, such as statutory health insurance providers, has mainly been used for this purpose to date (2). For data-driven medical research for specific projects and questions, the Informed Consent is an essential requirement for the use of electronic health records (EHRs) (3). In Germany, patients must be informed about the purpose, type of use, risks, and benefits associated with the data utilization before these data are allowed to be used for research (4). However, not all possible uses of the data are always foreseeable at the time consent is given. Therefore, a more generally formulated and thus more comprehensive wide consent, the so called Broad Consent (BC), would make sense.

As this study is situated in German primary care, it is important to note that primary care is predominantly delivered in an outpatient setting. These physicians usually work in small or medium-sized practices and act as the first point of contact for most health concerns, coordinating long-term and comprehensive care. This decentralized outpatient structure differs from more centralized or hospital-based systems in other countries and is particularly relevant for the implementation of BC, as recruitment and consent processes are strongly shaped by patient–physician relationships and practice organization (5).

Broad Consent is based on the Federal Data Protection Act (6) and the General Data Protection Regulation (GDPR) Article 6(1)(a) (7, 8). It has the advantage of allowing the collection, storage and secondary use of patient data for research purposes without the need for specific consent for each individual study (9). In addition, BC provides researchers with comprehensive access to a wide range of data, from demographic information to clinical outcomes and treatment information. The development of a uniform BC procedure for Germany by the Medical Informatics Initiative in 2020 was a milestone for the integration of EHR research (10). This standardized procedure is currently used at university hospitals and in the outpatient sector (10–12). In the database used for this study (see Methods), data are collected *via* a dual-track model that balances availability and data protection: fully anonymized datasets, where direct identifiers are irreversibly removed, are routinely extracted for descriptive analyzes, while patients who provide BC enable the use of pseudonymized datasets managed *via* a trusted third party. This explains why both anonymized and pseudonymized data are referred to throughout this article.

In Germany, data collection may follow Opt-In approaches (e.g., BC) requiring explicit patient consent or, in certain contexts, Opt-Out mechanisms. At the European level, the European Health Data Space (EHDS) Regulation (EU) 2025/327 entered into force in March 2025, with most secondary-use provisions phasing in *via* implementing/delegated acts and Member-state built out over the next two to six years later (13). Under the EHDS, the secondary use of health data will generally rely on opt-out and public-interest legal bases [GDPR Articles 6(1)(e) and 9(2)(j)], with ongoing debate about consent design, governance, and public value (13, 14). Mapping and policy analyzes highlight heterogeneous readiness among Member States, signaling that national choices (e.g., when consent remains required) will shape re-contact, linkage and retention in practice (15). Alongside legal progress, commentaries stress the need to balance data solidarity/public benefit with individual rights and trust to achieve a durable social license (14). This highlights a fundamental difference between BC and EHDS: while BC requires explicit Opt-In consent and ensures individual control, including the possibility of recontact for future studies, EHDS follows an Opt-Out model intended to increase and facilitate the availability and re-use of EHR data for public-interest purposes, while requiring secure processing environments, data minimization and (for any downloads) non-personal/anonymized outputs (GDPR Art. 6(1)(e), 9(2)(j) (14, 16). Accordingly, a detailed assessment of EHDS impacts lies outside the scope of this article, which focuses on BC in German general practice.

We hypothesize that obtaining BC during routine contacts in general practice preferentially captures patients with higher morbidity and greater healthcare utilization. Mechanistically, both self-selection (e.g., patients with chronic or life-limiting conditions being more motivated to contribute) and practice-driven processes (e.g., staff approaching patients who attend more frequently) could contribute to a systematic overrepresentation of older, multimorbid, and high-utilizing patients in the BC group compared with the broader practice population (17–22).

Selection biases may be an issue when BC is obtained as it was already reported for other types of consent in general practices (17–19, 23, 24). We have previously examined the differences in socioeconomic discrepancies between a BC group and a randomly selected control group within one general practice and found only small differences between the groups (25). For health and morbidity differences, data are scarce. It is known that consent to data use is higher among patients with lifelong or life-limiting illnesses (20). In addition, very large proportion of patients with rare diseases (97%) are in favor of using their data to better understand the disease and develop new treatments (26). This may be due to the fact that patients experience increased perceived self-efficacy through participation (27). When it comes to the general willingness to donate health data to science, healthy people show just as much (28) or even more (29) willingness as sick people. On the other hand, in a Swiss study, patients with comorbidities were more likely to consent to the use of their data after hospitalization than non-comorbid patients (30). Within the current investigation, the primary objective of this study is to compare health related parameters between patients who provided BC and the general practice population.

## Materials and methods

This study followed the Strengthening the Reporting of Observational Studies in Epidemiology (STROBE) guidelines for cross-sectional studies (31).

### Database

BeoNet-Halle represents a network of observational practices in primary care in Germany. From the practices, patient data is systematically collected, either anonymized or pseudonymized, and uploaded into a database for medical research purposes. Specifically, BeoNet-Halle follows a dual-track model: (i) routine extraction of fully anonymized datasets for exploratory and descriptive analyzes, and (ii) availability of richer pseudonymized datasets only for patients who provided BC; pseudonymization and consent status are administered by a trusted third party (see Ethical approval). The database is set up and maintained by the Institute of Medical Epidemiology, Biometry and Informatics and the Institute of General Medicine of the Medical Faculty of the Martin Luther University Halle-Wittenberg. BeoNet-Halle uses its own consent form which is based on the nationally harmonized BC documents of the Medical Informatics Initiative (10, 12, 32). No biosamples are obtained and utilized at BeoNet Halle. Patients are given various options for consent, for instance, to allow their patient data to be linked across participating practices and healthcare facilities or by permitting future re-contact.

The procedure for obtaining BC is carried out orally by the general practitioner or a specially trained member of the practice in accordance with §630e of the German Civil Code (BGB) on the duty to inform (33). After clarification of any remaining questions and sufficient time for reflection, the BC form is presented to the patient for review and signature. The study phase saw a switch from paper-based consent to electronic consent *via* tablet. Once a patient gives consent, the HL7 FHIR-compliant (Health Level 7^®^-Standard Fast Healthcare Interoperable Resources^®^) consent form is automatically transmitted to a trusted third party and fully processed electronically (34). The consent or revocation status is manually entered into the electronic patient record. Subsequently, the patient data are transferred pseudonymously from the practice management system to the BeoNet-Halle database, where they are made available for research.

So far, 11 solo and joint practices with a total of 44 general practitioners and 112,799 patients allowed the use of anonymous data. Of those, 473 patients agreed to BC.

### Study population

Patients were recruited in one BeoNet joint practice including eight general practitioners (GPs) between February 2021 and May 2023. The medical assistants asked for BC from patients who visit the practice for an appointment with the GP. All patients aged 20 and older who had a physician-patient contact with one of the participating GPs within the year 2022 were included. This so-called yearly contact group (YCG) is a way of calculating the denominators of primary care practices that stably represents the basic (35) practice population of patients (32, 35). A physician-patient contact is characterized by any instance in which an entry related to claims, such as reimbursement codes, medications, diagnoses, or referrals, is recorded in the practice management system (36). Two groups were defined from the 2022 YCG:

The first group called BC are patients which agreed to BC and which had a physician-patient contact in year 2022. The second group is the general reference population with at least one physician-patient contact during that period.

### Data extraction

We retrieved patient records from the BC-group and the RP within the BeoNet-Halle database for the YCG spanning January 1, 2022, to December 31, 2022. The extracted data encompassed a spectrum of information, including sociodemographic details, dates of physician-patient contacts, acute and chronic conditions, prescriptions, and referrals. This comprehensive dataset serves as the foundation for our subsequent analyzes of healthcare dynamics.

### Data analysis

The patient population, stratified by sex, is presented with counts and percentages. Age groups, physician-patient contacts, contact intervals, diagnoses and procedures are presented with means and standard deviation.

No inferential statistical measures or significance tests were applied since no random selection was drawn. Comparisons between the BC and the RP were carried out using aggregated data at patient level. This involved determining the proportion of patients who received at least one of the diagnoses, prescriptions and referrals examined during the study period. In addition, the number of multimorbid patients – in the present analysis defined as having more than 2 different chronic diseases during the study period (37) – was determined in both groups. Effect sizes (risk difference [RD] and Cohen’s w for categorical data, mean difference [MD] and η^2^ for metric data) were calculated to assess the differences between the BC group and the RP group. For Cohen’s w, values of 0.1, 0.3, and 0.5 are considered small, moderate, and large effects, respectively (38). For η^2^ values of 0.02, 0.06, and 0.14 are considered indicative of small, moderate, and large effects, respectively (38). To provide a more detailed understanding of healthcare dynamics, the ten most common acute confirmed acute diagnoses and the ten most common Anatomical Therapeutic Chemical (ATC) substance prescriptions in the YCG are presented with their relative frequency. *A priori*, we hypothesized that patients providing BC would, relative to the reference population, be older, have more physician–patient contacts, show higher prevalence of chronic conditions and multimorbidity, receive more referrals, and exhibit higher drug utilization, reflecting selection processes inherent to BC recruitment in routine care. All variables and comparisons were pre-specified: age (years), counts of physician–patient contacts, intervals between contacts (days), presence of at least one acute or chronic diagnosis (ICD-10), multimorbidity (>2 chronic conditions during 2022), referrals by specialty group, and prescriptions (ATC). Acute diagnoses exclude chronic codes; referral categories are listed in Figure 5; the 10 most frequent acute diagnoses and ATC substances are shown by relative frequency.

Drug utilization was quantified using Defined Daily Doses (DDD) according to the WidO ATC/DDD Index, aligned with the WHO ATC/DDD system (39, 40). Prescriptions were mapped to ATC codes and analyzed at ATC level 2. We report DDD per 1,000 inhabitants over the observation year and the mean difference (MD) between BC and RP for the ten therapeutic subgroups with the largest volumes (Table 3), following established procedures (41). Missing data were rare and handled by listwise deletion; no imputation was performed. Approach rates (i.e., numbers contacted or declining) were not recorded, limiting quantification of recruitment gatekeeping.

The statistical analyzes were conducted using Python 3.9 in conjunction with the Pandas library for robust data manipulation and analysis.

### Ethical approval

The study obtained ethics approval from the Martin-Luther-University Halle-Wittenberg’s researcher ethics committee (reference number: 2023-010). Ethical approval allowed the researchers to collect pseudonymized health and morbidity relevant data from patients who provided BC. From the RP data was collect and analyzed completely anonymized.

## Results

### Sample characteristics

During the study period, 5,034 patients had at least one physician-patient contact with one of the eight participating GPs (BC group: 439 patients, RP group: 4,595 patients). Sex was similar distributed between the two groups (Table 1). While patients in the BC group tended to be older, with a particular overrepresentation of 8.0 percentage points in the 70–79 years age group, based on effect sizes, none of these differences appear to be relevant.

**TABLE 1:** Sociodemographic variables (sex, age, insurance type) between RP group (*n* = 4,595) and BC group (*n* = 439).

	Reference population	Broad consent group	RP vs. BC	
	*n* (%)/Mean ± SD	*n* (%)/Mean ± SD	RD/MD	w/η^2^
**Number**				
	4,595	439		
**Sex**				
Female	2,363 (51.4%)	231 (52.6%)	1.2 pp	<0.01
**Age distribution**				
Mean	54.1 ± 18.8 (*n* = 4,593)	56.2 ± 17.0	2.1 years	<0.01
**Age groups**				
20–29	507 (11.0%)	32 (7.3%)	3.7 pp	0.03
30–39	727 (15.8%)	61 (13.9%)	1.9 pp	0.02
40–49	683 (14.9%)	54 (12.3%)	2.6 pp	0.02
50–59	745 (16.2%)	77 (17.5%)	1.3 pp	0.01
60–69	899 (19.6%)	106 (24.2%)	4.6 pp	0.03
70–79	522 (11.4%)	85 (19.4%)	8.0 pp	0.07
80–89	427 (9.3%)	21 (4.8%)	4.5 pp	0.04
90 or older	83 (1.8%)	3 (0.7%)	1.1 pp	0.02

Data is shown as count in numbers and frequencies (%) or as means with standard deviation. Risk difference (RD), mean difference (MD), η^2^ and Cohen’s w refer to the comparison between BC group and RP.

### Physician-patient contacts

Patients in the BC group had a higher number of physician–patient contacts with the participating general practitioners (15.0 vs. 9.2) and shorter intervals between physician contacts (28.7 vs. 34.3 days) than patients in the RP group (Table 2). Moreover, substantial differences were observed in that patients in the BC group more frequently had acute conditions (RD = 9.6 pp) and particularly chronic conditions (RD = 24.5 pp) than patients in the RP group. Proportionally more patients in the BC group than in the RP group were multimorbid (RD = 25.9 pp).

**TABLE 2:** Physician contacts per patient within a year, physician contact intervals and number of diagnosis per patient between RP group (*n* = 4,595) and BC group (*n* = 439).

	Reference population	Broad consent group	RP vs. BC	
	*n* (%)/Mean ± SD	*n* (%)/Mean ± SD	RD/MD	w/η^2^
**Physician contacts per patient**				
Mean	9.2 ± 10.8 (*n* = 4,595)	15.0 ± 12.5 (*n* = 439)	5.8	0.02
**Physician contact interval (days; only patients with at least two contacts within study period)**				
Mean	34.3 ± 40.8 (*n* = 3,355)	28.7 ± 29.9 (*n* = 420)	5.6	<0.01
**Patients with at least one diagnostic code (acute or chronic)**				
Acute (*n*)	3,935 (85.6%)	418 (95.2%)	9.6 pp	0.08
Chronic (*n*)	2,373 (51.6%)	334 (76.1%)	***24.5 pp***	***0.14***
**Multimorbid patients (more than 2 distinct chronic conditions during study period)**				
Multimorbid patients	1,659 (36.1%)	272 (62.0%)	***25.9 pp***	***0.15***

Data is shown as means with standard deviation. Risk difference (RD), mean difference (MD), η^2^ and Cohen’s w refer to the comparison between BC group and RP. Bold values highlight the most pronounced differences between groups.

### Chronic conditions

**FIGURE 1:**
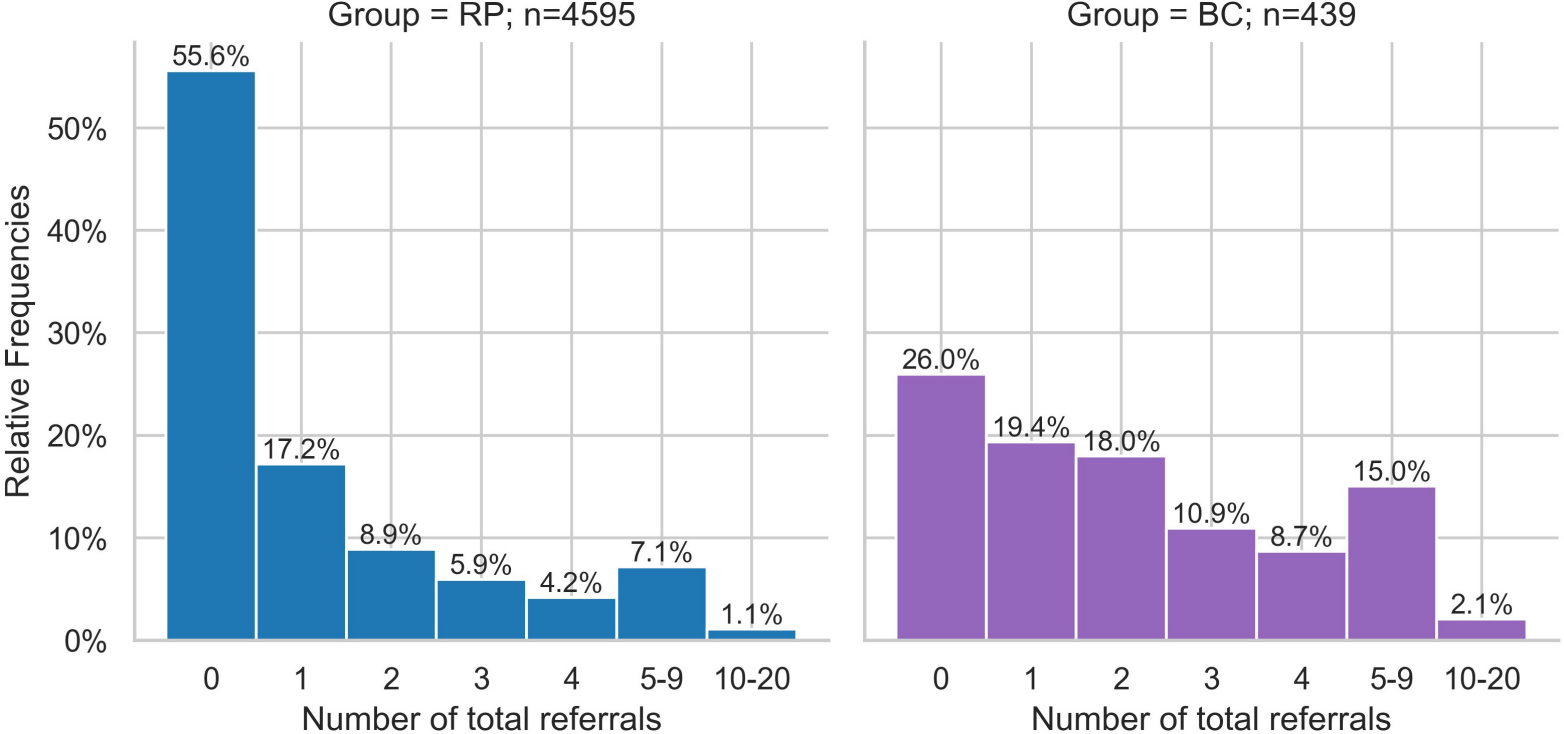
Relative frequency of patients with at least one diagnosis among the ten most frequent chronic conditions in Broad Consent Group (*n* = 439) and Reference Population (*n* = 4,595). Only effect sizes for *w* > 0.07 are marked.

**FIGURE 2:**
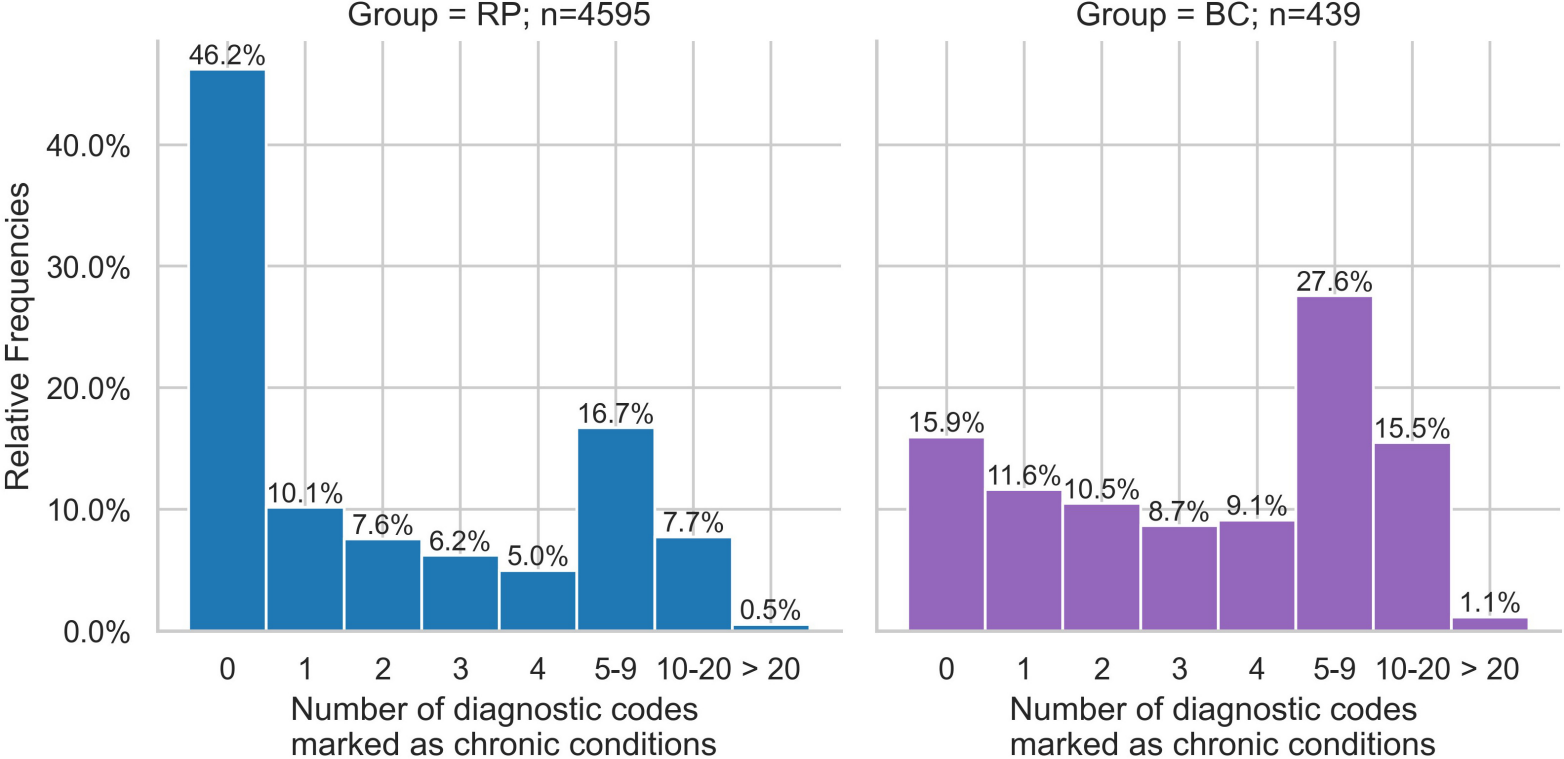
Relative frequencies of patients with number of chronic conditions of Broad Consent Group (*n* = 439) and Reference Population (*n* = 4,595).

The proportion as well as the order of chronic conditions was largely similar in both groups. However, the chronic conditions of essential hypertension (w = 0.09), dorsalgia (w = 0.09) and type 2 diabetes mellitus (w = 0.07) were proportionally more common in the BC group (Figure 1). Furthermore, the RP group had a higher percentage of individuals with no chronic conditions (46.2%) compared to the BC group (15.9%) (Figure 2). Conversely, the BC group exhibited a higher percentage of individuals with 5–9 chronic conditions (27.6%) compared to the RP group (16.7%). Overall, the distribution in the RP group was skewed toward fewer diagnoses, with notable peaks at 0 and 5–9 diagnoses, while the BC group had a more even distribution across the 1–9 diagnoses range, with a distinct peak at 5–9 diagnoses.

### ICD-codes excluding chronic conditions

**FIGURE 3:**
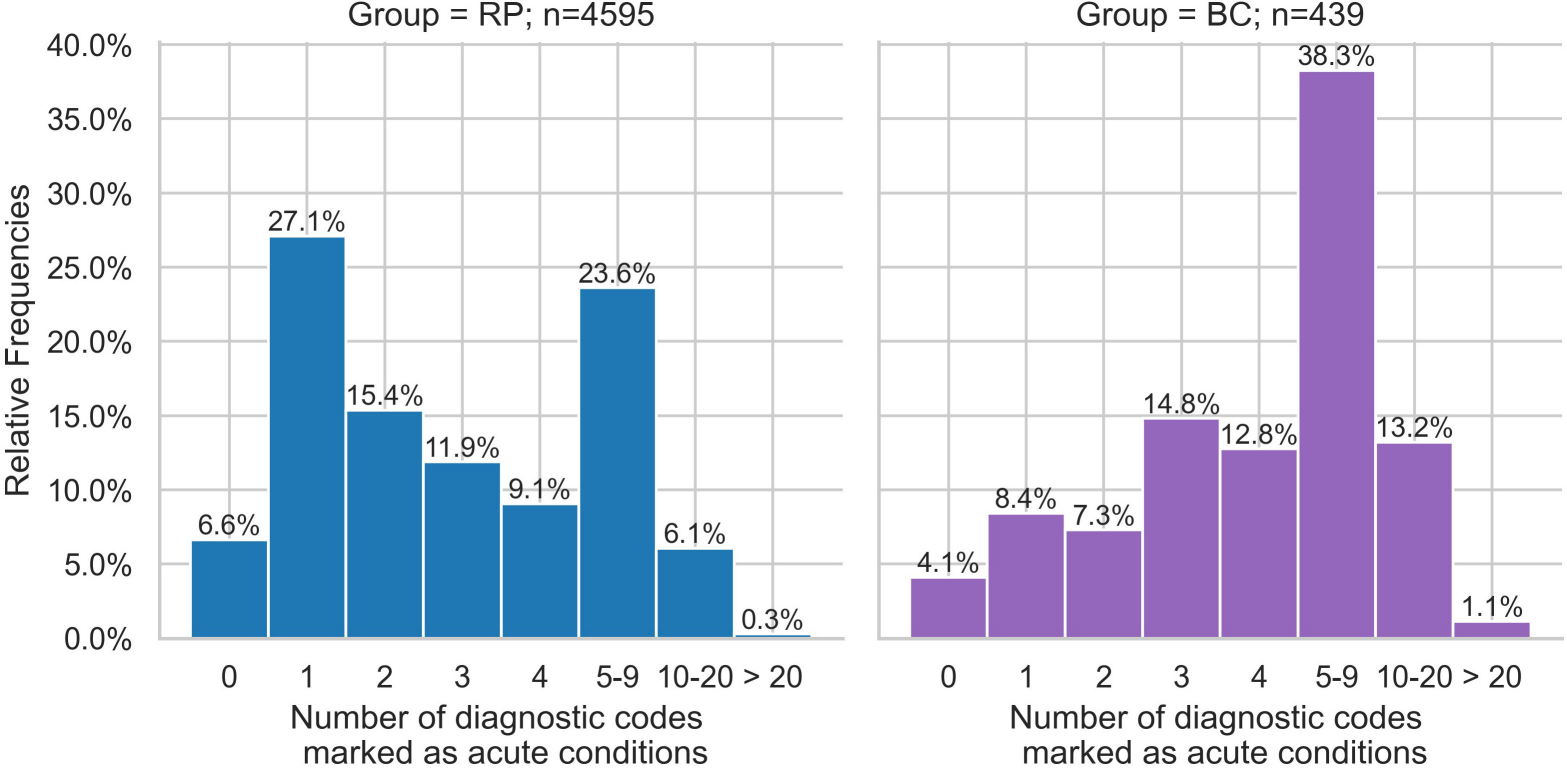
Relative frequency of patients with at least one diagnosis among the ten most frequent acute conditions in Broad Consent Group (*n* = 439) and Reference Population (*n* = 4,595). Only effect sizes for *w* > 0.07 are marked. Z01 code represents encounter for other special examination without complaint, suspected or reported diagnosis, such as hearing screenings or laboratory tests. U07 codes are to be used by WHO for the provisional assignment of new diseases of uncertain ethology. In GP practices they are normally used for identified or not identified COVID-19.

**FIGURE 4:**
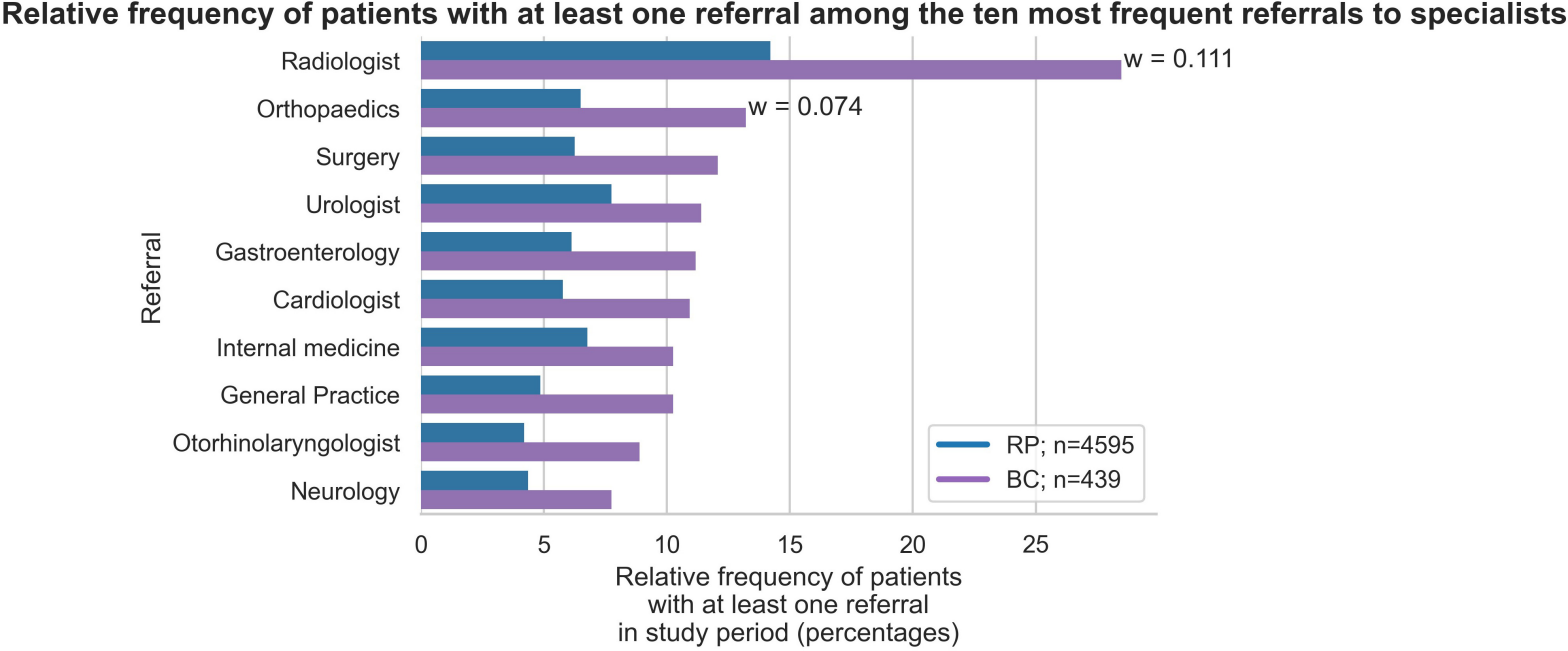
Relative frequencies of patients with number of chronic conditions of Broad Consent Group (*n* = 439) and Reference Population (*n* = 4,595).

Next, we examined the top 10 most frequent ICD-10 codes from all ICD chapters (A-Z), excluding codes for chronic conditions. These chapters include, for instance, codes on acute illnesses as well as reasons for encounters (ICD-10 Z-codes) and codes for special purposes (ICD-10 U-codes). More patients within the BC group had at least one encounter for repeat prescriptions (w = 0.129), general examinations (w = 0.095), and other special examinations without complaint (w = 0.104) (Figure 3). Although there were other differences between the BC and RP groups with regard to acute upper respiratory tract infections, reactions to severe stress and adjustment disorders, and other and unspecified infectious diseases, these differences showed small effect sizes. In terms of relative frequencies of acute conditions, encounters for repeat prescriptions were more common in the BC group (6.9 vs. 6.3%), while acute upper respiratory tract infections were more common in the RP group (3.2 vs. 2.4%). The RP group showed that the highest percentage of individuals had 1 acute condition (27.1%), followed by 5–9 diagnoses (23.6%) (Figure 4). In contrast, the BC group exhibited a peak at 5–9 diagnoses (38.3%), which was substantially higher than any other category.

### Medical measures and medication

**FIGURE 5:**
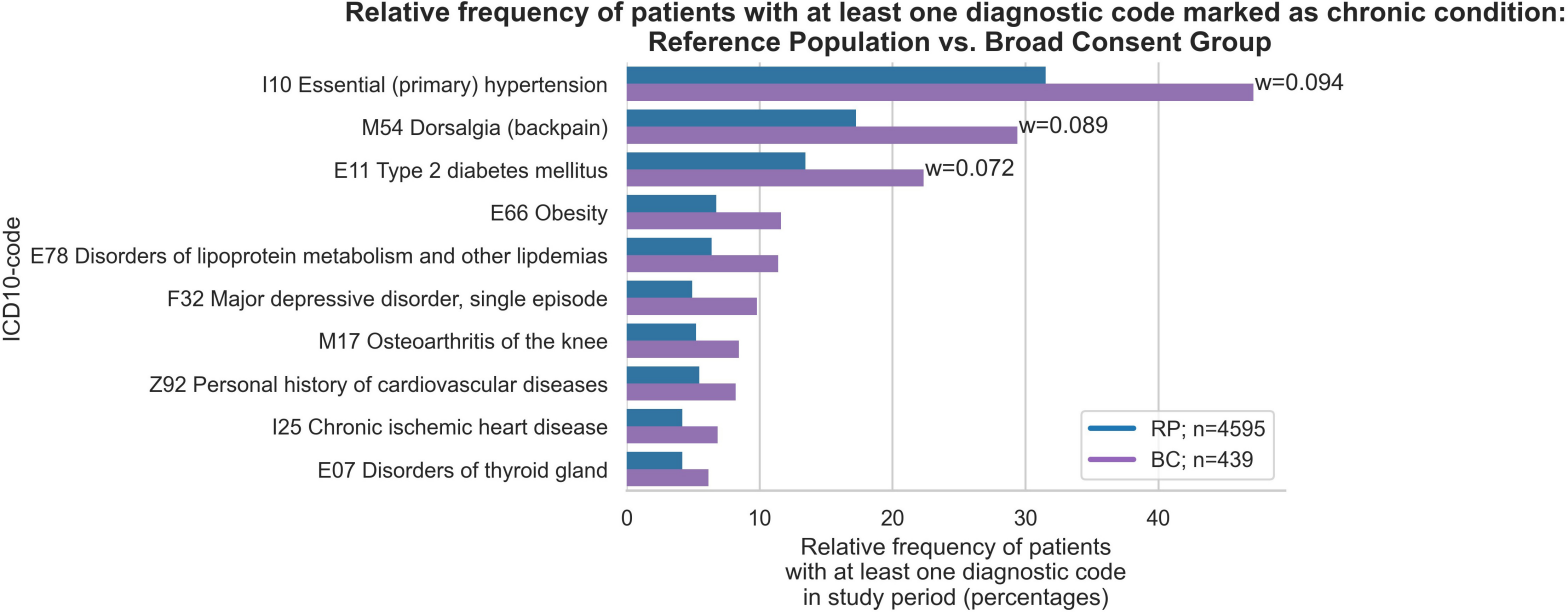
Relative frequency of patients with at least one referral among the ten most frequent referrals to specialists in Broad Consent Group (*n* = 439) and Reference Population (*n* = 4,595). Only effect sizes for *w* > 0.07 are marked.

**FIGURE 6:**
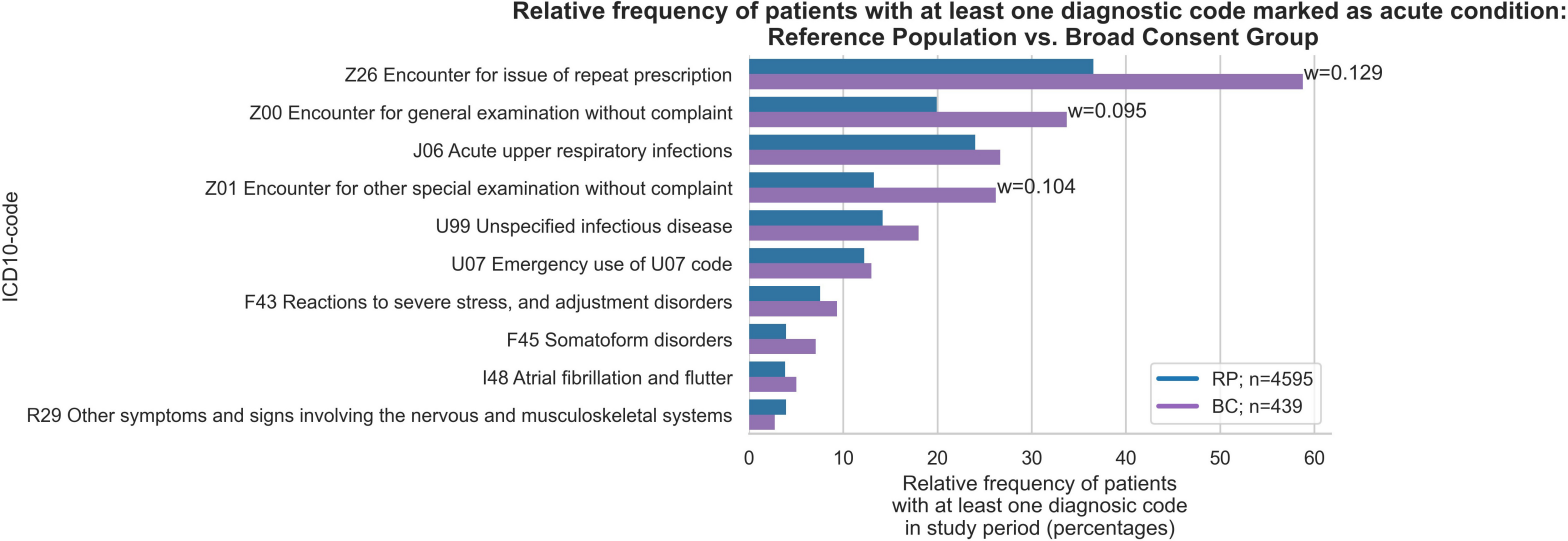
Relative frequencies of patients with number of total referrals of Broad Consent Group (*n* = 439) and Reference Population (*n* = 4,595).

During the study period, 74.0% (*n* = 325) of patients in the BC group and 44.4% (*n* = 2,040) of patients in the RP group received at least one referral to another physician in private practice (excluding referrals for laboratory diagnostics; w = 0.17; Figure 5). There were also significant differences in referrals for laboratory tests, with 72.9% (*n* = 320) of the BC group receiving a referral compared to only 48.5% (*n* = 2,229) of the RP group (w = 0.14). As shown in Figure 5, the differences between the BC and RP groups were particularly relevant for referrals to radiology (w = 0.11) and orthopedics (w = 0.07). The number of relative frequencies of patients with the total number of referrals shows that the number of patients in the BC group with 2 or more referrals is consistently twice as high as in the RP group (Figure 6).

The comparison of DDD per 1,000 inhabitants between BC and RP across the top 10 most frequent ATC subgroups revealed differences in drug prescription patterns (Table 3). Notably, drugs for acid-related disorders, diuretics and agents acting on the renin-angiotensin system had a markedly higher mean DDD per patient in BC compared to RP. Across all top 10 therapeutic subgroups, the BC group had a higher drug utilization.

**TABLE 3:** Comparison of Defined Daily Doses (DDD) per 1,000 Inhabitants between BC and RP across selected ATC Subgroups (sorted by BC).

ATC code	ATC subgroup name	BC Mean DDD per patient	RP Mean DDD per patient	BC – RP MD
A02	Drugs for acid related disorders	53.3	27.2	26.1
C03	Diuretics	47	26.1	20.9
C09	Agents acting on the renin-angiotensin system	44.3	26.2	18.1
C10	Lipid modifying agents	34.3	19.4	14.9
A10	Drugs used in diabetes	30.7	16.4	14.3
B01	Antithrombotic agents	30	20.9	9.1
C07	Beta blocking agents	29.7	17.1	12.6
A09	Digestives, including enzymes	13.5	7	6.5
N06	Psychoanaleptics	13.1	9.9	3.2

## Discussion

### Summary of the main findings

Patients in the BC group were older, had more physician-patient contacts, more acute and chronic conditions, and more referrals compared to the RP. Substantially more patients in the BC group were multimorbid than in the RP. More patients in the BC group than in the RP group received at least one prescription for a medication during the study period. The calculation of the DDDs revealed an overall higher drug consumption in the BC group than in the RP group.

### Discussion of the main findings

These patterns align with our working hypothesis of selection bias at the point of BC recruitment in routine practice. Effect sizes for chronic conditions (w≈0.14), multimorbidity (w≈0.15), and referrals (up to w≈0.17) indicate practically relevant differences rather than random fluctuation. This is consistent with prior evidence that informed-consent cohorts tend to include patients with greater comorbidity and disease burden, and that consent patterns vary with health status (20–22). Taken together, the findings suggest that BC-based outpatient datasets may systematically overrepresent sicker and higher-utilizing patients, which has implications for external validity when estimating prevalence or utilization from BC cohorts alone.

Previous results of age and consent are ambivalent. Both older and younger age may be associated with non-consent or consent (42, 43). In our study, we didn’t find a relevant effect of age on consent, which is also represented in research (17). However, depending on the setting and the type of medical research, age may be a relevant factor for consent decisions, for instance with decreasing consent rates for medical research projects being observed with increasing age (44).

In our study, the BC group had more physician-patient contacts than the RP group. This could be because the patients in the BC group were generally older and sicker. It is known that older people with more chronic conditions, especially multimorbid older people, have more frequent physician contacts than younger people (45). Frequent physician contacts could have a positive effect on the physician-patient relationship and thereby increase the willingness of patients to consent to BC. Patients with multiple conditions and therefore more frequent physician contacts enhanced their engagement in collaborating with the doctor on further treatment steps (46). More commitment to one’s own treatment may also be associated with a higher commitment to medicine in general and increasing willingness to participate. There is little research on higher comorbidities and greater multimorbidity. In line with our findings, a systematic review found that patients who gave informed consent had more comorbidities, including a higher overall comorbidity index, than patients who did not give consent (24). It is possible that patients with a higher burden of disease are more likely to recognize the benefit of their health data for medical research due to personal treatment benefits, and it is also conceivable that multimorbid patients may want to give something back to the treating physician by consenting (20). Therefore, patients’ burden of disease may be an influencing factor in consent, as medical staff may be more likely to approach or persuade patients with higher morbidity to participate. This is consistent with previous research showing that patients with a higher comorbidity index are more likely to provide consent (21, 24), and that country-level variations in consent often reflect health status and disease severity (20, 22). Additionally, practice staff may be more inclined to present a BC form to a patient they see frequently, whereas they may refrain from doing so for a brief, casual doctor-patient contact.

Consistent with the higher proportion of diagnoses and comorbidities in patients in the BC group, the drug consumption of these patients was higher. The large differences in consumption, especially for diuretics and drugs for acid related disorders, again indicate a higher proportion of chronically ill patients in the BC Group (47).

The results of the study indicate a selection bias that may arise during the selection and contacting of patients in the general practice. Patient groups with a low disease burden and infrequent doctor visits are underrepresented in the BC group. Thus, the results suggest that recruiting patients at the counter by nurses for the sensitive donation of health data with the BC may be an insufficient approach to addressing the German problem of making comprehensive patient data from real-world healthcare available for research. Possible reasons for this include the workload for nurses. The practice organization requires a high level of effort, which may cause nurses to prefer approaching easily recruitable patients. Patients who visit the practice less frequently, because they are younger and have fewer chronic conditions, are hardly reached and are rarely approached. Additionally, during the recruitment period, general practices were still affected by the organizational impacts of the COVID-19 pandemic, which may have also led to reduced recruitment by the nurses.

Our study does not allow for a definitive attribution of the selection bias to either self-selection (e.g., motivated patients with higher morbidity) or practice-driven mechanisms (e.g., staff preferentially approaching frequent attenders). However, it is important to emphasize that the existence of both types of bias has different implications for the design of future BC consent procedures. If self-selection dominates, motivational and educational strategies targeted at healthy or low-utilizing patients might be appropriate. If professional selection dominates, recruitment workflows need structural changes, such as delegating recruitment to trained staff independent of routine operations.

### Strengths and limitations

Our study highlights an important issue with high relevance for future research in the outpatient setting. To date, there is little data that specifically examines health and morbidity variables as possible factors influencing decision making in BC. The robustness of the results is supported by the large number of patients with *N* = 5,034 (BC *n* = 439, RP *n* = 4,595) who participated. The general sample of general practice patients allows a comparison with the BC group.

Although the patient-level sample is large, the practice-level sample size is effectively one joint practice with eight GPs, which limits representativeness and precludes any adjustment for practice-level clustering. Analyzes were descriptive without age–sex standardization or multivariable adjustment. As outcomes rely on routine EHR coding and ATC/DDD assignment, misclassification and missingness cannot be excluded. Approach rates (how many eligible patients were contacted or declined) were not recorded, which prevents quantifying gatekeeping. Recruitment was embedded in routine operations and initiated at the front desk by medical staff, so gatekeeping effects and workflow-dependent selection cannot be ruled out and may differ across organizations. Moreover, the study period still overlapped with pandemic-related organizational strain, and language barriers likely reduced outreach to non-German-speaking patients. Because no random sampling was performed and analyzes were descriptive by design, residual selection and information bias may remain; consequently, external validity beyond comparable German group practices should be inferred with caution.

### Implications for practice and further research

The results of this study suggest the need to implement the BC-consent procedure in general practices with targeted recruitment strategies to prevent selection bias in the selection and contacting of patients. Whether the selection bias stems from an increased interest in research among more severely ill patients or whether the practice staff are more likely to address patients who are frequently present in the practice is highly relevant for the time being. Due to the selection bias identified in this study, we have decided that in future surveys the research team will recruit the patients themselves in the practices. In doing so, we will systematically ensure that every patient - regardless of age, state of health, frequency of visits or language - is actively approached. If this cannot be fully implemented due to organizational or time constraints, previously underrepresented patient groups are specifically identified and prioritized in order to achieve a more balanced reflection of the reality of care.

The implementation of such active, structured recruitment measures is crucial in order to counteract the bias that has been proven to date and to provide valid, representative data for future healthcare research. This implementation requires further research and validation.

To disentangle self- from practice-driven selection and to improve generalizability, future studies should be conducted across multiple practices, stratified by practice type and region. Recruitment processes should be documented systematically, including approach rates and reasons for non-participation, to quantify gatekeeping effects. Importantly, medical assistants/receptionists, who often operationalize recruitment in daily routines but are underrepresented in research, should be explicitly included. Depending on feasibility, different recruitment modalities (e.g., research-team-led vs. practice-staff-led) could be compared, and analytic strategies such as weighting by visit frequency may help to mitigate bias.

## Conclusion

In this single-practice study, BC patients differed systematically from the broader practice population, with higher morbidity and utilization, consistent with prior evidence on consent patterns by health status (20–22). These differences were small-to-moderate in magnitude and likely reflect a mix of self-selection and practice-driven recruitment, implying that BC-based outpatient datasets may overrepresent sicker, higher-utilizing patients. While this can be advantageous for studies focusing on chronic disease, it limits external validity for descriptive epidemiology and service metrics. Given the single-site and descriptive design, the findings should not be used for population-level prevalence or utilization estimates without adjustment or sensitivity analyzes (e.g., age–sex standardization, weighting by visit frequency). Our findings therefore caution against relying on BC-only datasets for population-level prevalence or utilization estimates without safeguards. EHDS-related changes to consent workflows may alter selection patterns, but operational details remain uncertain at this stage.

## Data Availability

The original contributions presented in this study are included in this article/supplementary material, further inquiries can be directed to the corresponding author.
